# A promising ultra-sensitive CO_2_ sensor at varying concentrations and temperatures based on Fano resonance phenomenon in different 1D phononic crystal designs

**DOI:** 10.1038/s41598-023-41999-1

**Published:** 2023-09-12

**Authors:** Abdulkarem H. M. Almawgani, Hamza Makhlouf Fathy, Hussein A. Elsayed, Yahya Ali Abdelrahman Ali, Ahmed Mehaney

**Affiliations:** 1 Electrical Engineering Department, College of Engineering, Najran University, Najran, Kingdom of Saudi Arabia; 2 Physics Department, Faculty of Science, Beni-Suef University, Beni-Suef, 62512 Egypt; 3 Information Systems Department, College of Computer Sciences and Information Systems, Najran University, Najran, Saudi Arabia

**Keywords:** Engineering, Materials science, Physics

## Abstract

Detecting of the levels of greenhouse gases in the air with high precision and low cost is a very urgent demand for environmental protection. Phononic crystals (PnCs) represent a novel sensor technology, particularly for high-performance sensing applications. This study has been conducted by using two PnC designs (periodic and quasi-periodic) to detect the CO_2_ pollution in the surrounding air through a wide range of concentrations (0–100%) and temperatures (0–180 °C). The detection process is physically dependent on the displacement of Fano resonance modes. The performance of the sensor is demonstrated for the periodic and Fibonacci quasi-periodic (S_3_ and S_4_ sequences) structures. In this regard, the numerical findings revealed that the periodic PnC provides a better performance than the quasi-periodic one with a sensitivity of 31.5 MHz, the quality factor (Q), along with a figure of merit (FOM) of 280 and 95, respectively. In addition, the temperature effects on the Fano resonance mode position were examined. The results showed a pronounced temperature sensitivity with a value of 13.4 MHz/°C through a temperature range of 0–60 °C. The transfer matrix approach has been utilized for modeling the acoustic wave propagation through each PnC design. Accordingly, the proposed sensor has the potential to be implemented in many industrial and biomedical applications as it can be used as a monitor for other greenhouse gases.

## Introduction

Nowadays, using gas sensors to monitor interior and outdoor CO_2_ emissions is a major goal to achieve effective environmental preservation. Among other gases, CO_2_ is a greenhouse gas, which is released into the environment^1^. The most frequent ones include ozone, CO_2_, methane, nitrous oxide, and sulfur dioxide^2^. Significant amounts of CO_2_ are emitted into the atmosphere because of human activity, which has exceeded the standard threshold limit. Since the nineteenth century, global warming has become a serious issue in every country worldwide. Numerous human activities have resulted in an unceasing release of greenhouse gases into the atmosphere, which, in turn, raised the earth’s temperature to an alarming level^3^. Besides, very high concentrations of CO_2_ have led to the global warming phenomenon. Therefore, the natural carbon cycle is disrupted by global warming, which has been increasing the released amount of CO_2_ into the atmosphere. Consequently, the continuance of the carbon cycle has considerably affected the earth’s environment^1–5^. Moreover, burning fossil fuels, land degradation, deforestation, and other detrimental industrial practices significantly increased, leading to releasing higher levels of CO_2_ into the atmosphere^4^. This has, in turn, caused a global concern due to the negative effects on the climate change and environment as well^1–5^. Moreover, increased CO_2_ emissions have accelerated the greenhouse effect, thereby increasing global warming levels by 0.8 °C in 2013^6–9^. This can result in damaging effects on the environment, thereby melting ice caps, raising sea level, causing severe weather conditions, and so on^10,11^. Additionally, CO_2_ emissions have caused air pollution because of its greenhouse effect, trapping radiation and producing the ozone layer at a ground level; the atmospheric layer of the ozone inhibits the cooling of the earth at night^12–14^. Another consequence is ocean waters warming as they can absorb CO_2_ from the atmosphere^13^. Consequently, this has resulted in a higher water temperature, thereby affecting the ability of the oceans to absorb CO_2_. The negative effects of CO_2_ emission will be significantly exacerbated over time^15,16^. Another environmental CO_2_ impact on the surrounding air pollution is the existence of the climate change phenomenon as the surface of the earth is currently warmer; this phenomenon has existed over the last one hundred years, as per research conducted by NOAA, i.e., the National Oceanic and Atmospheric Administration^17–20^. Furthermore, CO_2_ has led to an additional environmental impact, which is the acid rain phenomenon. Emissions of gas from power plants that burn fossil fuels are mixed with air moisture, causing precipitation with higher acid content^21–24^. Evidence has demonstrated substantial physical damages to trees and plants. Soil and water pollution has occurred because of acid rain. Gas emissions’ movement is an additional factor, in addition to the CO_2_ emission impact as they are felt and observed far from sources. This has, in turn, made the CO_2_ effects on air pollution more serious^25^. CO_2_ emissions affect human health, relocating oxygen in the atmosphere, thereby breathing has become more and more difficult. When the CO_2_ level rises, especially in enclosed areas, higher amounts of CO_2_ has led to many health complaints, for example, headaches^26–29^. Additionally, CO_2_ levels have exhibited a high level of dangerous air pollutants, for example, unstable organic compounds, which trigger indoor air contamination^27,30–33^. Many physical and chemical sensing devices are available for CO_2_ detection purposes. For instance, several sensors have been constructed to detect CO_2_, including catalytic, fluorescent, semiconductor thin films, and optical gas sensors^4,34–37^. However, these methods and techniques may collide with the high fabrication cost, limited performance, difficulty in fabrications and limited accuracy as well. Therefore, the need for some new models and design with a limited cost, high performance, good stability, and ease during fabrication becomes mandatory for this purpose.

Meanwhile, phononic crystals, i.e., (PnCs) are regarded as artificial periodic structures, which are composed of two or more materials. These materials vary in mass density and elastic properties, as well as sound speed^38–42^. The PnCs composite structures can be utilized for the greenhouse gas sensing because of the acoustic speed of sound in the gaseous mixture can vary based on its composition^41,43^. Therefore, the PnC are considered appropriate acoustic gas sensors, which are used for low-cost and experimental sensing applications without the lead time^38–40^. Thus, acoustic waves are transmitted and reflected at the components’ interfaces. The wave interference consequently happens at each of the layers’ interface. Depending on the incident acoustic waves’ frequency, the periodic arrangement causes both constructive and destructive interferences^38–40^. Due to completely destructive interference, the PnCs structures can exhibit the stop bands for the waves at certain frequencies called (PnBGs), i.e., phononic band gaps^42,44,45^. The acoustic waves cannot pass through the structure within these frequency ranges. The PnBG condition can be established similar to Bragg condition. Regarding electromagnetic waves, the band gap is comparable to what happens in photonic crystals’ periodic structures. Thus, the optical and acoustic waves’ properties are the main investigated topics in the physics of photonic crystals and PnCs, respectively^44,46^. The photonic and PnCs’ unique characteristics can provide remarkable applications depending on a wide variety of designs. There are a lot of applications for the PnCs-BG properties, including mechanical filters, ultrasonic imaging systems, noise suppression, sensors, and acoustic diodes^47–50^. Furthermore, one advantage of employing PnCs is characterized by their flexibility to regulate external influences on the reference, as well as target gases, such as pressure or temperature^51,52^. In addition, the PnCs structure with a cavity inserted inside that is filled with various gases provides key advantages compared with the conventional PnCs. When a cavity is added inside a PnCs structure, for example, the structure’s periodicity is broken and multiple resonance frequencies are generated through the PnCs-BnPG, which adds to the uniqueness of the PnCs structures compared with the periodic ones^53,54^. Recently, sensor technology has been growing in significance as an important field of study for numerous modern technological applications. The PnCs particularly attracted sensor researchers’ interest because they are efficient, flexible, demonstrating reliable materials with longer expectancy concealing^38–40^. The PnCs-based devices are thought to be the best option for creating acoustic gas sensors. Kushwaha is the first scholar to introduce PnCs structures, showing their capability of controlling the mechanical waves to capture, send, or stop propagation at certain frequencies^55^. For instance, Zubtsov et al. used the two dimensional (2D) PnCs with a resonant cavity to measure the amount of ethanol in gasoline^56^. Additionally, a sensitive biosensor, which measures the temperature of a biomaterial (methyl nonafluorobutyl ether) in a broad range 10–40 °C utilizing a 2D triangular lattice solid/fluid PnCs, is demonstrated^57^.

Additionally, many previous studies focused on using the PnCs as gas sensors. For instance, Ahmet et al. examined the sensors of CO_2_ depending upon the 1D surface acoustics PnCs computationally and experimentally^58^. Mehaney investigated the design of a porous PnC sensor, theoretically as a specific CO_2_ gas sensor sensor depending upon a one-dimensional (1D) porous silicon (PSi) PnCs placed between a couple of thin layers of rubber. The development of a CO_2_ gas sensor has, therefore, become necessary^59^. Human daily lifestyles are filled with many artificial and natural oscillators and resonators, starting from lasers to sophisticated systems, such as imaging machines and musical instruments. The introduction of Fano resonance method inside PnBG for PnCs sensor structures has been the focus of attention because they have sharp, asymmetric line shapes^60,61^. They were first introduced over fifty years ago. Since then, Fano resonance has received a lot of attention because of their sharp and asymmetric line shapes produced by a destructive interference between the states of narrow discrete and the states of the large continuum^61,62^. This has drawn a lot of attention due to the wide variety of sensors and optical devices to which it can be applied. Fano resonance has been investigated in the PnCs periodic structures and were utilized in the PnCs structure acoustic waveguide systems^60–64^. The Fano resonance is used in a lot of phononic applications, including PnCs resonators, radiation detectors, and waveguiding. Periodic/quasi-periodic PnCs gas sensors’ structure with the Fano resonance phenomenon have not been explored before as it is believed that this produces very sharp modes of resonance transmitted with an innovative sensitivity, the quality factor, and the figure of merit^60–64^. The quasi-periodic structures are known as structures of an exceptional arrangement pattern, which provides extra design freedom and controls the properties of the structure; these structures lack translational symmetry^65–69^. To create omnidirectional band gaps, as well as large phononic/photonic band gaps, these quasi-periodic structures could function more efficiently compared to the periodic designs. Solid–solid and solid–fluid structures have so far been created in 1D and 2D PhCs with quasi-periodic structures^65,68,70,71^. The available literature about the 1D or the 2D PnCs sensors does not include the Fano resonance phenomenon. Cicek et al. experimentally studied a new acoustic gas sensor but without the implementation of Fano resonance modes. They have also developed a new acoustic gas sensor utilizing the PnCs for the CO_2_ gas. The interaction of an acoustic wave with a gas has also been proposed by Cheeke et al.^38,58^.

Therefore, this study investigates the sensing of CO_2_ concentration in air depending upon the periodic/quasi-periodic PnCs and by using the transfer matrix method (TMM), the transmission spectra can be calculated. Slow and sharp Fano modes are demonstrated in the PnBG for each gas concentration and temperature value. The introduced Fano modes through the periodic/quasi-periodic PnCs structures considered an innovative sensing tool as it shifts with the finest change in the gas concentration. Moreover, this study discusses the effects of varying temperature on the Fano resonance modes’ position that generated through the periodic, S_3,_ and S_4_ quasi-periodic PnCs. The projected 1D-DPnCs gas sensor is constructed easily theoretically and experimentally because of using the 1D multilayered structures in various sensor applications. The introduced gas sensor in this study contains low-cost materials like lead and epoxy, and it can function under demanding conditions like high pressures and temperatures. Furthermore, it does not involve complicated electric components. This study also examines the effects of varying temperatures on sensitivity, the quality factor, and the figure-of-merit for the periodic, S_3_, and S_4_ quasi-periodic PnCs gas sensor based on quite a sharp mode of the Fano resonance.

## Materials and theoretical formulization

### PnCs sensor structure

**Figure 1:**
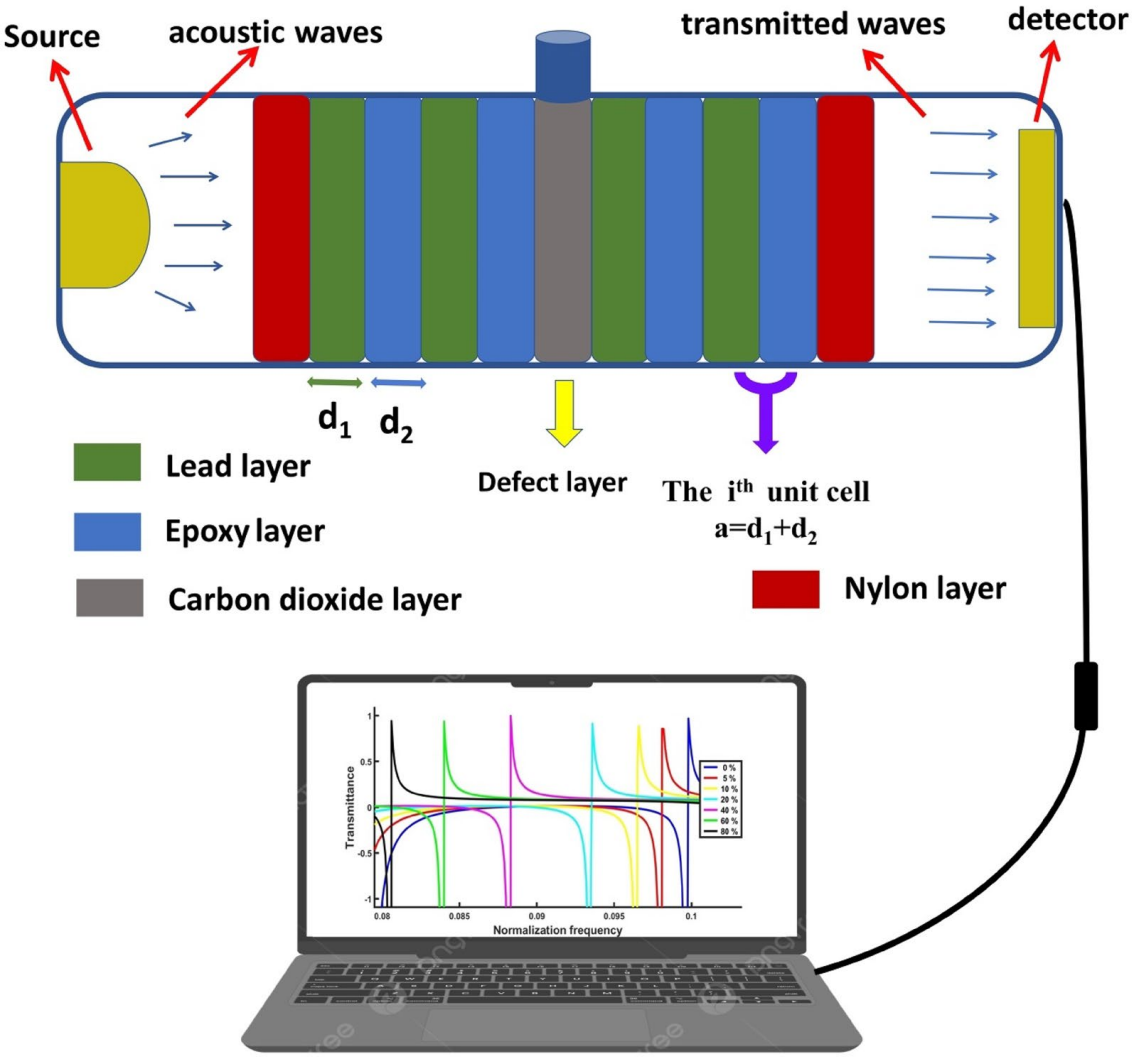
Schematic diagram for the 1D-PnC gas sensor’s structure, comprising a given periodic multilayer of epoxy and lead using a CO_2_-filled defect layer.

**Figure 2:**
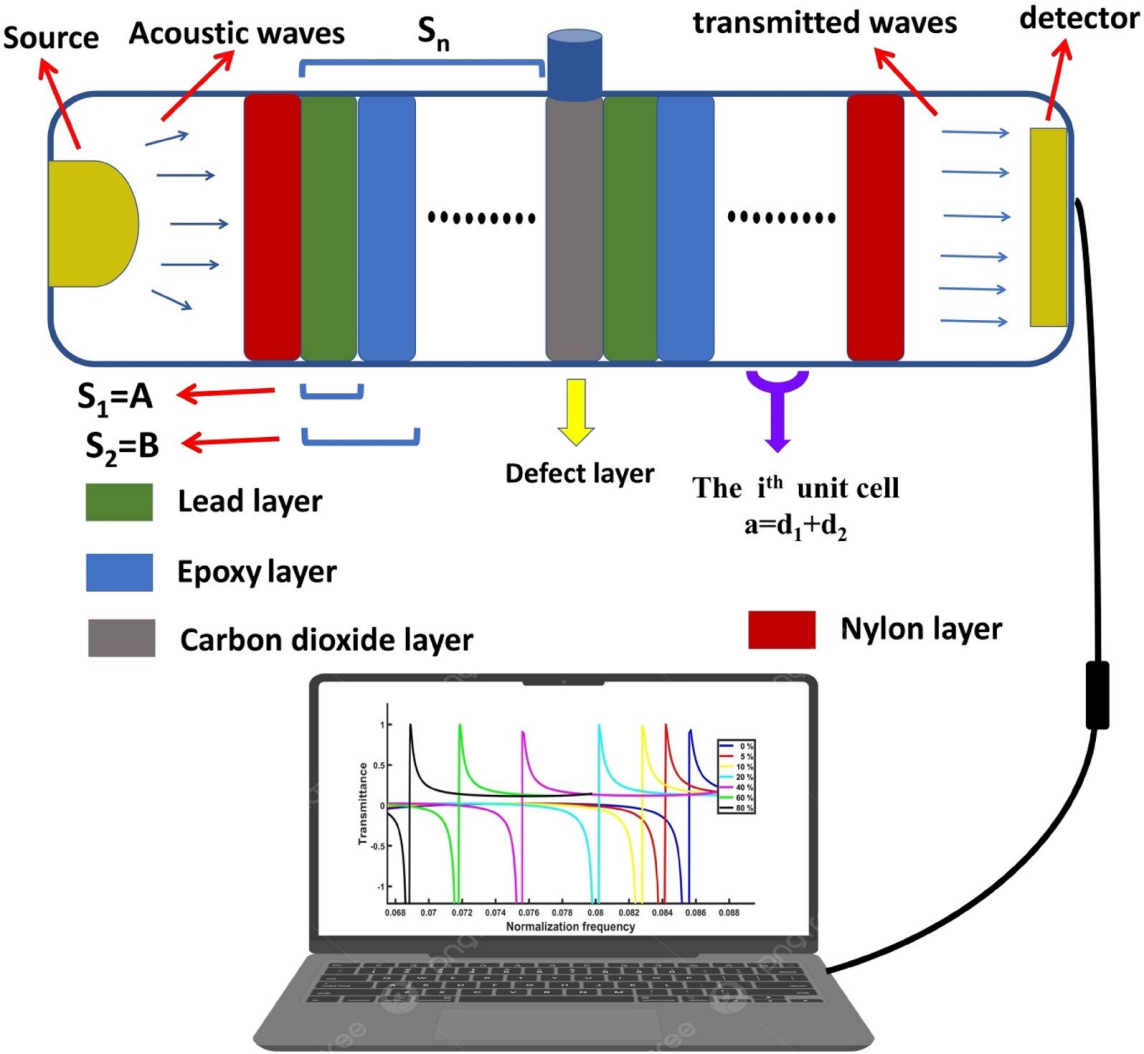
Schematic diagram for the projected 1D-PnCs Fibonacci quasiperiodic sensor structure of S_n_/CO_2_ gas/S_n_.

In this work, the periodic, as well as the quasi-periodic PnC structures are presented as a CO_2_ gas sensor by means of utilizing the mode of Fano resonance in PnBG. The periodic PnC structure consists two diverse layers of Lead/Epoxy repeated in N = 4-unit cells with equal thicknesses, followed by inserting a defect layer in the middle of the structure i.e., [(Lead/Epoxy)^2^ − (CO_2_ gas) − (Lead/Epoxy)^2^]. The lattice constant or the length of each unit cell is given as *a* = *d*_1_ + *d*_2_, where *d*_1_ and *d*_2_ define the thickness of the first and second layers, respectively, as shown in Fig. 2. The thicknesses of Lead and Epoxy were proposed as 0.1 µm and 0.1 µm, respectively, in all calculations. The operating frequency has been adjusted within the ultrasonic range. The filled defect layer with carbon dioxide has been added in the center of these structures. The construction materials’ acoustic properties are displayed in Table 1. Lead and epoxy have a high acoustic mismatch, which enables the generation of a broad PnBG. Consequently, the propagated waves are distributed in this structure at an interface between each of the two utilized layers, and when this interference is a constructive one, it will form PnBG. In contrast, a transmission band is formed if the interference is destructive^44,45,59^. The constructed layers’ acoustic properties with the given gas-filled layer represent parameters, which are considered input variables and used to illustrate its attempt at a gas sensor. As displayed in Fig. 1, the PnC structure is periodicity arranged, so its layers, acoustic properties, such as density and acoustic speed, varied regularly.

**Table 1:** Values for the construction materials’ acoustic properties.

Material	Density (kg/m^3^)	Longitudinal speed C_L_ (m/s)	Thickness (μm)
Lead	10,760	1960	1
Epoxy	1140	2770	1
Air (reference)	1.2047	343 1	1.5

Another proposed model of the quasi-periodic 1D PnCs structure is constructed from a specific multilayer stack, consisting of two materials: epoxy and lead. The quasi-periodic PnCs structure is designed according to the stacking rule, which is covered in the analyzed structures section. The structures of quasi-periodic lack translational symmetry, introducing periodic structures of extraordinary ordering patterns, and providing an extra freedom in constructing and controlling the characteristics of the structure^69,71–74^. As a result, the acoustic wave’s ability to propagate through these structures is increasingly attenuated because of the additional degree of freedom it has within them^65,71–74^. The proposed visualization of the quasi-periodic PnCs design is [ABADABA] and [ABAABDABAAB], where (A) describes lead, and (B) describes epoxy. The D layer, which is imposed to be surrounded by Fibonacci quasi-periodic sequences, is the CO_2_ gas. The defect layer of CO_2_ gas is imposed to be surrounded by Fibonacci quasi-periodic structures from both sides to study the quasi-periodic PnCs structure as a gas sensor to CO_2_.

## Theoretical treatment

### Transfer matrix method

In this study, the binary structures PnC gas sensor is designed, as presented in Figs. 1 and 2. Periodic, as well as the quasi-periodic PnC structures directed considerable attention because they perform efficiently in sensing applications^65,75–77^. Inside the multilayer PnC structures, acoustic sound waves are transmitted and reflected. To explore the acoustic waves’ transmission using the PnCs, several approaches were developed, including the TMM, i.e., the transfer matrix method, the plane wave expansion (PWE) method, and the finite difference time domain (FDTD) method^65,75–77^. Nonetheless, among all these methods, only the transfer method may be used to determine how the incident acoustic waves can be transmitted using the given multilayer structure^78^.

At intersections between the structure’s layers, the ${d}_{j}$ indicates the thickness of layer along the $x$-axis. The condition of continuity for acoustic waves’ propagation were considered. If the given acoustic wave enters the introduced multilayer PnC structure, it will disperse into multiple waves within the structure, thereby changing the acoustic properties of the layer, including wave speed and density periodically. The propagation of the acoustic wave in the proposed multilayer gas sensor PnCs structures, as shown in Figs. 1 and 2, can be established by using the following differential equation^79,80^.

1 $$\frac{1}{{\mathrm{C}}_{\mathrm{j}}^{2}}\frac{{\partial }^{2}\mathrm{p}}{\partial {\mathrm{t}}^{2}}-{\nabla }^{2}\mathrm{p}=0,$$

where C_i_ refers to the sound speed (longitudinal speed) in layer j, subscript j refers to the number of the layers inside the PnCs structure, and p refers to the pressure of the acoustic wave. Thus, the preceding equation solution is demonstrated by this equation:

2 $${\mathrm{p}}_{\mathrm{j}}=\left({\mathrm{A}}_{+}^{(\mathrm{j})}{\mathrm{e}}^{+{\mathrm{iK}}_{\mathrm{j}}\mathrm{X}}+{\mathrm{A}}_{-}^{(\mathrm{j})}{\mathrm{e}}^{-{\mathrm{iK}}_{\mathrm{j}}\mathrm{X}}\right){\mathrm{e}}^{\mathrm{i\omega t}},$$

where ${\mathrm{A}}_{+}^{(\mathrm{j})}$ and ${\mathrm{A}}_{-}^{(\mathrm{j})}$ refer to amplitudes of the forward (transmitted) and backward (reflected) waves’ respectively, $\upomega$ refers to the propagation waves’ angular frequency, and ${\mathrm{K}}_{\mathrm{j}}=\upomega /{\mathrm{C}}_{\mathrm{j}}$ refers to the wave vector, which differs according to the waves’ acoustic sound speed across the layers of the structure.

There is a continuity for the wave displacement and applied stress should be observed at the boundary between every two layers following how acoustic waves interact with the proposed structures. The generated stress by the acoustic waves over these introduced structures is shown by this equation^81–83^:

3 $$\upsigma ={\mathrm{E}}_{\mathrm{j}}\frac{\partial {\mathrm{p}}_{\mathrm{j}}}{\partial \mathrm{x}},$$

where ${\mathrm{E}}_{\mathrm{j}}$ indicates Young’s modulus of any material used to construct the PnCs structure. The stress can be displayed by computing Eqs. (2) and (3) as the following equation:

4 $$\begin{array}{cc}& \\ & \sigma (x)=i{Z}_{j}\left[{A}_{+}^{(j)}{e}^{+i{K}_{j}X}-{A}_{-}^{(j)}{e}^{-i{K}_{j}X}\right]\end{array},$$

where ${Z}_{j}={E}_{j}{K}_{j}$ refers to the acoustic impedance. Thus, the wave displacement $\mathrm{u}(\mathrm{x})$ and stress $\sigma (\mathrm{x})$ components can be written in the following matrix form as;

5 $$\left[\begin{array}{l}\mathrm{u}(\mathrm{x})\\ \sigma (\mathrm{x})\end{array}\right]=\left[\begin{array}{cc}1& 1\\ i{Z}_{j}& i{Z}_{j}\end{array}\right]\left[\begin{array}{c}{A}_{+}^{(j)}{e}^{+i{K}_{j}X}\\ {A}_{-}^{(j)}{e}^{-i{K}_{j}X}\end{array}\right]=\left[\begin{array}{c}{A}_{+}^{(j)}{e}^{+i{K}_{j}X}\\ {A}_{-}^{(j)}{e}^{-i{K}_{j}X}\end{array}\right],$$

where ${B}_{j}$ indicates the wave matrix at the boundary of two layers. With the help of these components, we may apply the relationship ${X}_{R}^{j}={X}_{L}^{j}+{d}_{j}$ where ${X}_{R}^{j}$ and ${X}_{L}^{j}$ indicate the left and right boundary position of every layer $(j$), respectively. Consequently, displacement, as well as stress are related from ${X}_{L}^{j}$ to those at ${X}_{R}^{j}$, as illustrated in the following equation:

6 $$\begin{array}{cc}& \left[\begin{array}{c}\mathrm{u}\left({\mathrm{X}}_{\mathrm{R}}^{\mathrm{j}}\right)\\\upsigma \left({\mathrm{X}}_{\mathrm{R}}^{\mathrm{j}}\right)\end{array}\right]=\left[\begin{array}{cc}{\mathrm{e}}^{+{\mathrm{iK}}_{\mathrm{j}}{\mathrm{d}}_{\mathrm{j}}}& 0\\ 0& {\mathrm{e}}^{-{\mathrm{iK}}_{\mathrm{j}}{\mathrm{d}}_{\mathrm{j}}}\end{array}\right]{\mathrm{B}}_{\mathrm{j}}\left[\begin{array}{c}{\mathrm{A}}_{+}^{(\mathrm{j})}{\mathrm{e}}^{+{\mathrm{iK}}_{\mathrm{j}}}{\mathrm{x}}_{\mathrm{L}}^{\mathrm{j}}\\ {\mathrm{A}}_{-}^{(\mathrm{j})}{\mathrm{e}}^{-{\mathrm{iK}}_{\mathrm{j}}{\mathrm{X}}_{\mathrm{L}}^{\mathrm{j}}}\end{array}\right]={\mathrm{P}}_{\mathrm{j}}{\mathrm{B}}_{\mathrm{j}}\left[\begin{array}{c}{\mathrm{A}}_{+}^{(\mathrm{j})}{\mathrm{e}}^{+{\mathrm{iK}}_{\mathrm{j}}{\mathrm{x}}_{\mathrm{L}}^{\mathrm{j}}}\\ {\mathrm{A}}_{-}^{(\mathrm{j})}{\mathrm{e}}^{-{\mathrm{iK}}_{\mathrm{j}}{\mathrm{X}}_{\mathrm{L}}^{\mathrm{j}}}\end{array}\right]\\ & {\text{And}} \; \left[\begin{array}{c}\mathrm{u}\left({\mathrm{X}}_{\mathrm{L}}^{\mathrm{j}}\right)\\\upsigma \left({\mathrm{X}}_{\mathrm{L}}^{\mathrm{j}}\right.\end{array}\right]={\mathrm{B}}_{\mathrm{j}}\left[\begin{array}{c}{\mathrm{A}}_{+}^{(\mathrm{j})}{\mathrm{e}}^{+{\mathrm{iK}}_{\mathrm{j}}{\mathrm{x}}_{\mathrm{L}}^{\mathrm{j}}}\\ {\mathrm{A}}_{-}^{(\mathrm{j})}{\mathrm{e}}^{-{\mathrm{iK}}_{\mathrm{j}}{\mathrm{x}}_{\mathrm{L}}^{\mathrm{j}}}\end{array}\right] \end{array}$$

The following equation indicates the propagation matrix across every layer (j), which defines the acoustic waves’ propagation through one layer $\mathrm{j}$ with a certain thickness ${\mathrm{d}}_{\mathrm{j}}$ of the introduced multilayer PnCs gas sensor structure^78^:

7 $${\mathrm{P}}_{\mathrm{j}}=\left[\begin{array}{cc}{\mathrm{e}}^{+{\mathrm{iK}}_{\mathrm{j}}{\mathrm{d}}_{\mathrm{j}}}& 0\\ 0& {\mathrm{e}}^{-{\mathrm{iK}}_{\mathrm{j}}{\mathrm{d}}_{\mathrm{j}}}\end{array}\right]$$

Therefore, Eqs. (6) and (7) are re-written as:

8 $$\left[\begin{array}{l}\mathrm{u}\left({\mathrm{X}}_{\mathrm{R}}^{\mathrm{j}}\right.\\\upsigma \left({\mathrm{X}}_{\mathrm{R}}^{\mathrm{j}}\right.\end{array}\right]={\mathrm{P}}_{\mathrm{j}}{\mathrm{B}}_{\mathrm{j}}\left[\begin{array}{c}{\mathrm{A}}_{+}^{(\mathrm{j})}{\mathrm{e}}^{{\mathrm{iK}}_{\mathrm{j}}{\mathrm{X}}_{\mathrm{L}}^{\mathrm{j}}}\\ {\mathrm{A}}_{-}^{(\mathrm{j})}{\mathrm{e}}^{-{\mathrm{iK}}_{\mathrm{j}}{\mathrm{X}}_{\mathrm{L}}^{\mathrm{j}}}\end{array}\right]={\mathrm{B}}_{\mathrm{j}}{\mathrm{P}}_{\mathrm{j}}{\mathrm{B}}_{\mathrm{j}}^{-1}\left[\begin{array}{c}\mathrm{u}\left({\mathrm{X}}_{\mathrm{L}}^{\mathrm{j}}\right)\\\upsigma \left({\mathrm{X}}_{\mathrm{L}}^{\mathrm{j}}\right)\end{array}\right]={\mathrm{D}}_{\mathrm{j}}\left[\begin{array}{c}\mathrm{u}\left({\mathrm{X}}_{\mathrm{L}}^{\mathrm{j}}\right)\\\upsigma \left({\mathrm{X}}_{\mathrm{L}}^{\mathrm{j}}\right)\end{array}\right],$$

where ${\mathrm{D}}_{\mathrm{j}}$ indicates the transfer matrix of layer $\mathrm{j}$. Thus, for a similar layer $\mathrm{j}$, we can relate Eq. (8) with the components of the displacement and stress at the left $\left({\mathrm{X}}_{\mathrm{L}}^{\mathrm{j}}\right)$ to right $\left({\mathrm{X}}_{\mathrm{R}}^{\mathrm{j}}\right)$, as illustrated in this equation^81^:

9 $${\mathrm{D}}_{\mathrm{j}}=\left[\begin{array}{cc}\mathrm{cos}\left({\mathrm{K}}_{\mathrm{j}}{\mathrm{d}}_{\mathrm{j}}\right)& 1/{\mathrm{Z}}_{\mathrm{j}}\mathrm{sin}\left({\mathrm{K}}_{\mathrm{j}}{\mathrm{d}}_{\mathrm{j}}\right)\\ -{\mathrm{Z}}_{\mathrm{j}}\mathrm{sin}\left({\mathrm{K}}_{\mathrm{j}}{\mathrm{d}}_{\mathrm{j}}\right)& \mathrm{cos}\left({\mathrm{K}}_{\mathrm{j}}{\mathrm{d}}_{\mathrm{j}}\right)\end{array}\right]$$

As the transfer matrix can be applied to any layer and ${\mathrm{X}}_{\mathrm{L}}^{\mathrm{j}}\equiv {\mathrm{X}}_{\mathrm{R}}^{(\mathrm{j}-1)}$, we can extend Eq. (9) to include many structural layers, as illustrated in the following equation:

10 $$\begin{array}{cc} &\mathrm{y}\left({\mathrm{X}}_{\mathrm{R}}^{1}\right) = {\mathrm{D}}_{1}\mathrm{y}\left({\mathrm{X}}_{\mathrm{L}}^{1}\right)=\mathrm{y}\left({\mathrm{X}}_{\mathrm{L}}^{2}\right),\mathrm{y}\left({\mathrm{X}}_{\mathrm{R}}^{2}\right)={\mathrm{D}}_{2}\mathrm{y}\left({\mathrm{X}}_{\mathrm{L}}^{2}\right)={\mathrm{D}}_{2}{\mathrm{D}}_{1}\mathrm{y}\left({\mathrm{X}}_{\mathrm{L}}^{1}\right)=\mathrm{y}\left({\mathrm{X}}_{\mathrm{L}}^{3}\right),\\ & \mathrm{y}\left({\mathrm{X}}_{\mathrm{R}}^{3}\right)={\mathrm{D}}_{3}\mathrm{y}\left({\mathrm{X}}_{\mathrm{L}}^{3}\right)={\mathrm{D}}_{3}{\mathrm{D}}_{1}{\mathrm{D}}_{2}\mathrm{y}\left({\mathrm{X}}_{\mathrm{L}}^{1}\right)=\mathrm{y}\left({\mathrm{X}}_{\mathrm{L}}^{4}\right)\\ & \mathrm{y}\left({\mathrm{X}}_{\mathrm{R}}^{\mathrm{n}}\right)={\mathrm{D}}_{\mathrm{n}}{\mathrm{D}}_{\mathrm{nl}}\cdots {\mathrm{D}}_{1}\mathrm{y}\left({\mathrm{X}}_{\mathrm{L}}^{1}\right)=\mathrm{Dy}\left({\mathrm{X}}_{\mathrm{L}}^{1}\right)\end{array}$$

Consequently, the matrix (D) can link stress and wave displacement at the left end $\left(X={X}_{L}^{j}\right)$ of the layer ($j$) in each unit cell to the right of the nth layer $\left(X={X}_{R}^{j}\right)$. The impedance ${Z}_{j}$ and ${E}_{j}$ of each material are used to design the PnCs structure and determine how the ${D}_{j}$ matrix behaves. Equations 11, 12, and 13 can be used to express components of the total transfer matrix ${D}_{ij}=D(i,j)$, as mentioned in Eq. (9):

11 $${D}_{j}(\mathrm{1,1})={D}_{j}(\mathrm{2,2})=\mathrm{cos}\left({K}_{j}{d}_{j}\right)$$

12 $${D}_{j}(\mathrm{1,2})=1/{Z}_{j}\mathrm{sin}\left({K}_{j}{d}_{j}\right)$$

13 $${D}_{j}(\mathrm{2,1})=-{Z}_{j}\mathrm{sin}\left({K}_{j}{d}_{j}\right)$$

As ${Z}_{j}={E}_{j}{K}_{j}$ refers to the impedance, and ${d}_{j}$ is the layer’s thickness. We can compute the coefficient of transmission of the proposed PnCs structure by using the following Equation^81^:

14 $$\frac{{U}_{e}}{{U}_{0}}=\frac{2{E}_{0}\left({D}_{11}{D}_{22}-{D}_{12}{D}_{21}\right)}{{E}_{0}\left({D}_{11}-{E}_{e}{D}_{21}\right)-\left({D}_{12}-{E}_{e}{D}_{22}\right)}$$

The ${U}_{0},{U}_{e}$ indicate the wave amplitudes of the incident and transmitted wave, respectively, whereas ${E}_{0}$ and ${E}_{e}$ refer to two semi-infinite solids Young’s moduli at the left and right of the $\mathrm{PnCs}$ structure.

### Structure analysis

In this section, acoustic wave propagation is introduced through the binary periodic and S_n_ quasi-periodic structures. The quasi-periodic PnCs structures in the present study can be presented by the Fibonacci sequence^69,84,85^. By placing the two basic components A and B side by side according to the recursive rule, as illustrated in Eq. (15), a Fibonacci structure can be produced experimentally.

15 $${\mathrm{S}}_{\mathrm{n}}={\mathrm{S}}_{\mathrm{n}-1}{\mathrm{S}}_{\mathrm{n}-2}, \; \mathrm{ for \; n}\ge 2$$

By starting with ${\mathrm{S}}_{0}=\mathrm{B}$ and ${\mathrm{S}}_{1}=\mathrm{A}$. So, the Fibonacci sequence is generated as

$${\mathrm{S}}_{2}=\mathrm{AB}; {\mathrm{S}}_{3}=\mathrm{ABA}; {\mathrm{S}}_{4}=\mathrm{ABAAB},\text{ etc}.$$

Therefore, a defective quasi-periodic PnC structure can be designed as a gas sensor by inserting a specific defect layer in the middle, i.e., between two sequences of layers, which have the same Fibonacci structure, as shown in Fig. 2. The S_3_ quasi-periodic PnCs structure is studied [$({\mathrm{S}}_{3}$)(CO_2_ gas)(${\mathrm{S}}_{3}$)], as well as the S_4_ of the quasi-periodic PnCs structure [$({\mathrm{S}}_{3}$)(CO_2_ gas)(${\mathrm{S}}_{3}$)], as shown in Table 2.

**Table 2:** Sequence of the layers for quasi-periodic PnCs structures.

Structure (S_n_)	Layer sequence
S_3_/CO_2_/S_3_	ABADABA
S_4_/CO_2_/S_4_	ABAABDABAAB

### Calculations CO_2_/air mixture

**Figure 3:**
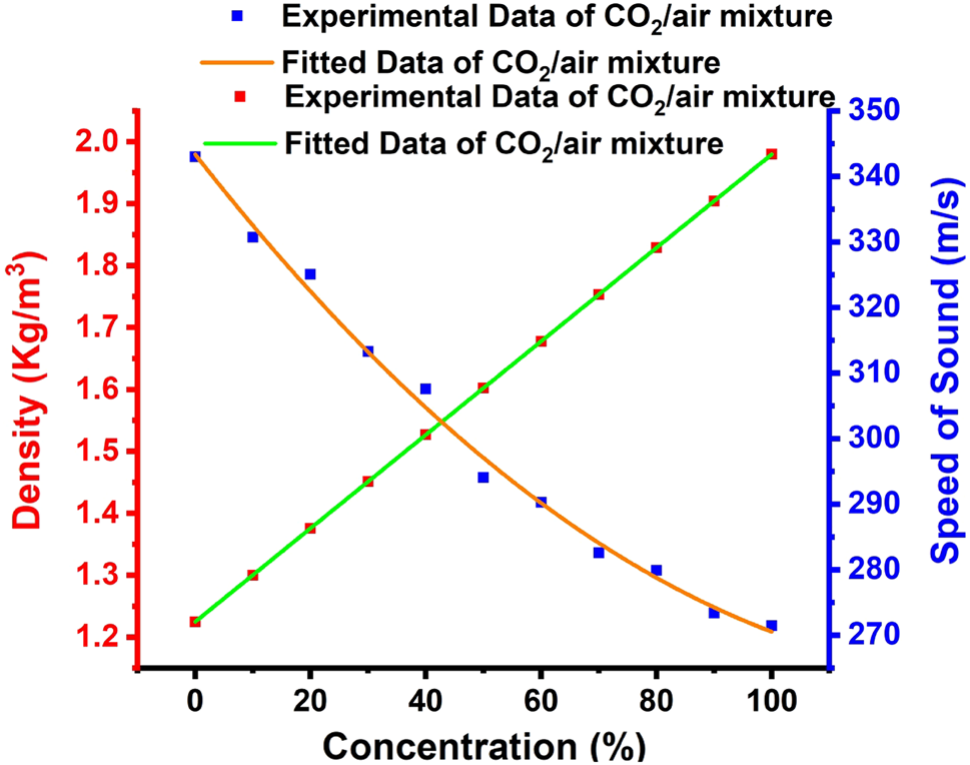
Density and speed of sound for the CO_2_-air mix as a certain function in the concentration.

All the calculations below are initially considered at sea level and atmospheric pressure. The densities of air and ${\mathrm{CO}}_{2}$ are 1.225 and $1.98 \, \mathrm{kg}/{\mathrm{m}}^{3}$ at 20 °C, respectively. The density of the air and CO_2_ mixture is computed as follows^86^:

16 $${\uprho }_{\mathrm{m}}={\uprho }_{1}{\mathrm{f}}_{1}+{\uprho }_{2}{\mathrm{f}}_{2},$$

where ${\uprho }_{\mathrm{m}},{\uprho }_{1}$, and ${\uprho }_{2}$ indicate the densities of the mixture, air, and ${\mathrm{CO}}_{2}$, respectively. ${\mathrm{f}}_{1}$ and ${\mathrm{f}}_{2}$ indicate the volume fraction of air and ${\mathrm{CO}}_{2}$, respectively. The following Eq. (17) can be used to calculate the relationship between the longitudinal sound speed and the gaseous mixture’s composition (air and ${\mathrm{CO}}_{2}$) for binary mixtures^87^:

17 $${\mathrm{c}}^{2}=\left(\frac{\mathrm{RT}}{{\mathrm{m}}_{1}\left(1-{\mathrm{X}}_{12}\right)+{\mathrm{m}}_{2}{\mathrm{X}}_{12}}\right)\left(\frac{{\mathrm{C}}_{\mathrm{pl}}\left(1-{\mathrm{X}}_{12}\right)+{\mathrm{C}}_{{\mathrm{p}}^{2}}{\mathrm{X}}_{12}}{{\mathrm{C}}_{\mathrm{v}1}\left(1-{\mathrm{X}}_{12}\right)+{\mathrm{C}}_{22}{\mathrm{X}}_{12}}\right),$$

where $\mathrm{T}$ indicates the temperature of the gas in Kelvin, $\mathrm{R}$ indicates the gas constant $\frac{8.314 \, \mathrm{J}}{\mathrm{mol}} {\mathrm{K}}^{-1}$, where ${\mathrm{m}}_{1}$ and ${\mathrm{m}}_{2}$ indicate the molecular weights of the two components in the mixture. ${\mathrm{C}}_{\mathrm{p}1},{\mathrm{C}}_{\mathrm{p}2},{\mathrm{C}}_{\mathrm{v}1}$, and ${\mathrm{C}}_{\mathrm{v}2}$ indicate specific heat capacity at constant pressure and volume in the two components, respectively,${\mathrm{where X}}_{12}$ indicate the partial pressure of component 1 in component $2.$ Figure 3 depicts the density and sound speed as a function of the concentration of ${\mathrm{CO}}_{2}$/air mixture. Because ${\mathrm{CO}}_{2}$ has a large density ($1.98 \, \mathrm{kg}/{\mathrm{m}}^{3}$) than air ($1.225 \, \mathrm{kg}/{\mathrm{m}}^{3}$), the density of the ${\mathrm{CO}}_{2}$/air mixture rises as the ${\mathrm{CO}}_{2}$ concentration rises, and because the speed of sound in air (343 m/s) is higher than that of ${\mathrm{CO}}_{2}$ (271.5 m/s), the speed of sound in the ${\mathrm{CO}}_{2}$/air mixture drops as the ${\mathrm{CO}}_{2}$ concentrations rise.

The effect of different concentrations of CO_2_ on the acoustic properties (i.e., sound velocity and density) is discussed depending upon the provided references. To generalize the given correlation between the concentrations and the acoustic properties, a numerical fitting for the provided data was considered. Thus, the experimental data regarding the CO_2_ mass density are fitted so that this equation is met as follows:

18 $$\rho \left(\frac{\mathrm{kg}}{{\mathrm{m}}^{3}}\right)=\alpha +b\times \complement \left(\mathrm{\%}\right),$$

where $\rho$ refers to the density, $\complement$ refers to the concentration, whereas $\alpha$ and $b$ represent constants of the fitted relation such that: $\alpha$ = 1.225 and $b$ = 0.00755. According to the preceding equation, there is a linear relationship between the density of CO_2_ gas and its concentration, as illustrated in Fig. 3. The gas’s density and concentration are connected. As a result, the density of a gas increases along with its concentration. The experimental data are then fitted to the following equation to obtain the CO_2_ sound speed:

19 $$v\left(\frac{m}{s}\right)={\upbeta }_{1}-{\upbeta }_{2}\times \complement (\mathrm{\%})+{\upbeta }_{3}\times ({\complement (\mathrm{\%}))}^{2},$$

where $v$ is the velocity of sound, $\complement$ defines its concentration, and ${\upbeta }_{1},{\upbeta }_{2} \; {\mathrm{ and}} \, \upbeta_{3}$ represent constants of a fitted relationship. Thus, the values of coefficients are provided:${\upbeta }_{1}=$ 343.39161 $, {\upbeta }_{2}=$ 1.12399 ${\mathrm{ and}}\; \upbeta _{3}=$ 0.00396. Figure 3 shows that sound velocity declines with an increased CO_2_ concentration, which results in a polynomial linear fitting based on the preceding equation.

## Results and discussion

### Transmission spectra of PnC designs

**Figure 4:**
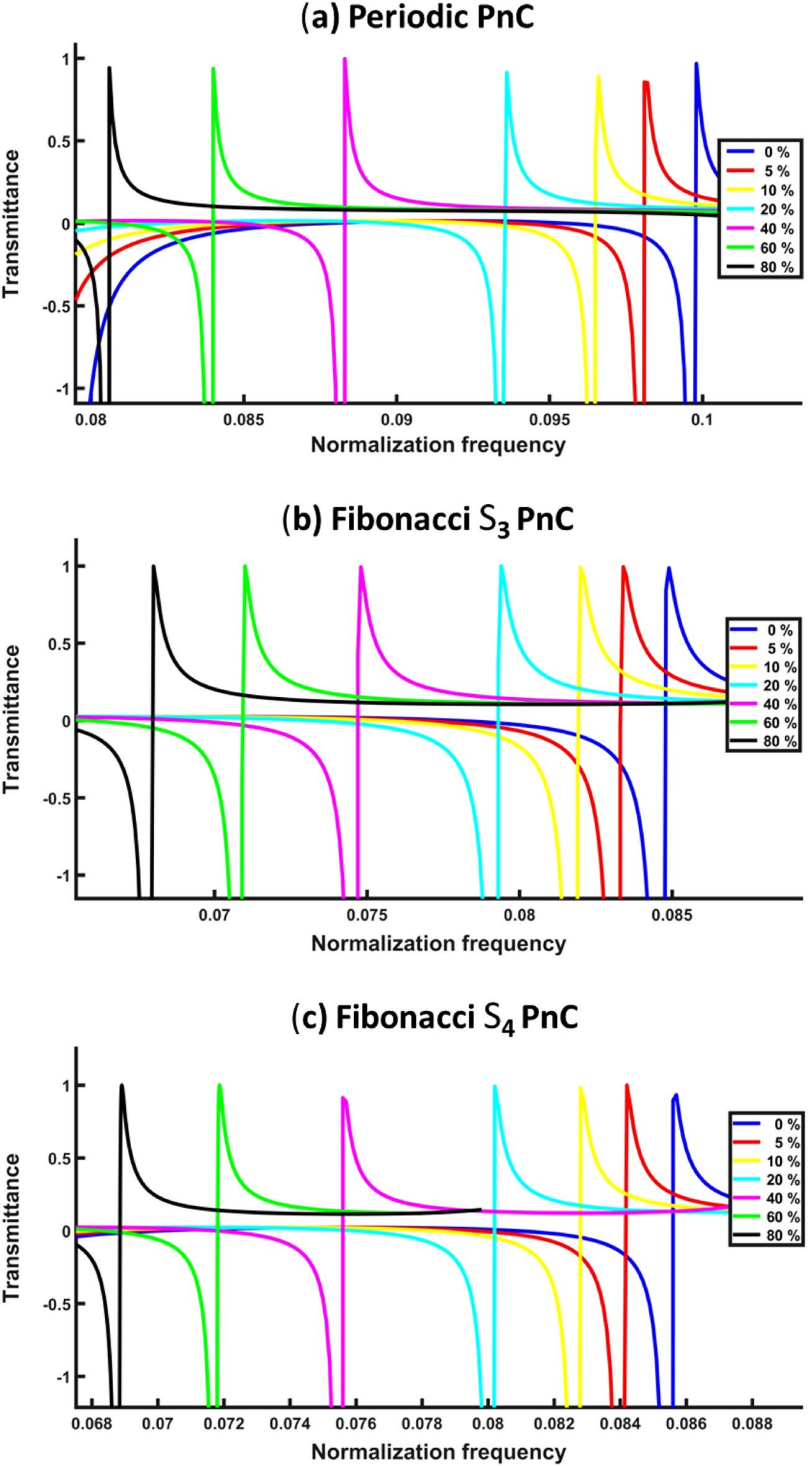
The Fano resonance transmitted spectra as opposed to the normalized frequency to the CO_2_ gas at room temperature of the following PnCs gas sensor structures, (**a**) periodic, (**b**) Fibonacci sequence S_3_ and (**c**) Fibonacci sequence S_4_.

This section debates the effects of increasing CO_2_ concentrations on the features of Fano resonance transmitted spectra as opposed to a normalized frequency inside the PnBG related to periodic, S_3_, and S_4_ PnC structures. The Fano resonance peak’s frequency and intensity change when CO_2_ concentrations change, as illustrated in Fig. 4. As observed, the Fano resonance peaks in the periodic PnC structure have a higher frequency than those in the other quasi-periodic PnCs structure. The proposed three PnC structures can detect the CO_2_ gas effectively according to these Fano resonance peaks. They can also show the precise quantity of the target CO_2_ gas, as well as the physical characteristics of the gas with remarkable sensitivity, quality, and figure of merit. According to the results, the periodic PnCs structure has the highest normalized frequency among various PnCs structures, followed by S_4_ and S_3_ for the quasi-periodic PnC structures. By applying to periodic PnC structures, CO_2_ showed Fano resonance peak with an 85% transmitted intensity at a value of a normalized frequency (0.0982) when a 5% concentration is used. Figure 4 shows that S_3_ quasi-periodic PnC structure provided Fano peak appeared at a frequency of 0.0842 and intensity of a 99% when a 5% concentration is used. Fano resonance peak of S_4_ quasi-periodic PnC structure is apparent at a normalized frequency value of 0.0834, transmitting intensity of approximately 99% when a 5% concentration is used. Figure 4 shows the broadest range of frequency in periodic, as well as the Fibonacci S_4_ quasi-periodic PnC structures compared with the Fibonacci S_3_ quasi-periodic PnC structure. This is due to the number of layers and interfaces are developed in the periodic, and S_4_ quasi-periodic higher than the S_3_ quasi-periodic PnCs structure, which, in turn, enhances the interference of acoustic waves at each interface and then the formation of wide band gaps and resonant mode as well. In addition, when the structure’s number of layers increased due to the disordered arrangement, there was a significant attenuation for acoustic sound waves in the structure. The Fano resonance, which appeared for every gas was examined. Fano resonance is a special type of resonance in the photonics, as well as phononics^87^, which result from a destructive interference through interaction between discrete and continuum states at the interface of the revealed layers^63,79,80^. Fano resonance peaks in this work are extraordinary because they have quite a sharp line shape with robust correlation with sensitivity measurements. Fano peaks were presented in the projected sensor design because the frequency of Fano resonance improves sensitivity, as well as the values of the quality factor^63,88^. Fano resonance peaks shift towards high frequencies when the acoustic speed for the sound of CO_2_ increases (see Fig. 3). Sensitivity with other sensor’s performance parameters for periodic, S_3_, and the S_4_ quasi-periodic PnCs structures to CO_2_ at room temperature as the resonance frequency function were calculated in the subsequent section.

### Sensor parameters

This section debates how the projected sensing device perform using several related measurements, such as sensitivity (S), quality factor (QF), a figure of merit (FOM), and damping rate (ζ). Here, we utilized MATLAB for conducting the computational and numerical analyses in this study. Additionally, Origin was employed for generating some of the graphical figures presented in this research. Furthermore, the utilization of MATLAB allowed us to implement complex algorithms and simulations, facilitating the in-depth investigation of the proposed periodic and quasi-periodic PnCs structures. The software's versatility and computational capabilities played a crucial role in analyzing the acoustic band gap characteristics and evaluating the sensor's sensitivity and performance. In conjunction with MATLAB, Origin provided an intuitive platform for visualizing and interpreting the obtained results. The generation of graphical figures using Origin enhanced the clarity and effectiveness of the research findings, enabling readers to grasp the key outcomes with greater ease. By combining the power of MATLAB's computational abilities and Origin's visualization capabilities, the authors were able to deliver a comprehensive and compelling study on the potential of phononic crystals as advanced gas sensors for CO_2_ detection. The synergy between these software tools strengthened the scientific rigor of the research, reinforcing the significance and reliability of the reported outcomes. These measurements are believed to be relevant for describing the sensor’s performance. The defect mode features have the most influence on their values^89–91^.

20 $$S=\frac{\Delta f}{\Delta C}$$

21 $$Q=\frac{{f}_{r}}{{f}_{HBW}}$$

22 $$\mathrm{FOM}=\frac{s}{{F}_{HBW}}$$

23 $$\zeta =\frac{1}{2*Q}$$

where S represents the Sensitivity and its units is in Hz. Δ $f$ denotes the change in resonant frequency, and its unit is in Hz. ΔC signifies the change in concentration, and its unit is in percentage (%). Q represents the Quality Factor, and it is a dimensionless quantity. ${f}_{r}$ denotes the frequency of the resonant peak, and its unit is in Hz. ${F}_{HBW}$ indicates the frequency of the half bandwidth of the given peak, and its unit is in Hz. FOM represents the Figure of Merit, and it is a dimensionless quantity. ζ represents the Damping Factor, and it is a dimensionless quantity.

**Figure 5:**
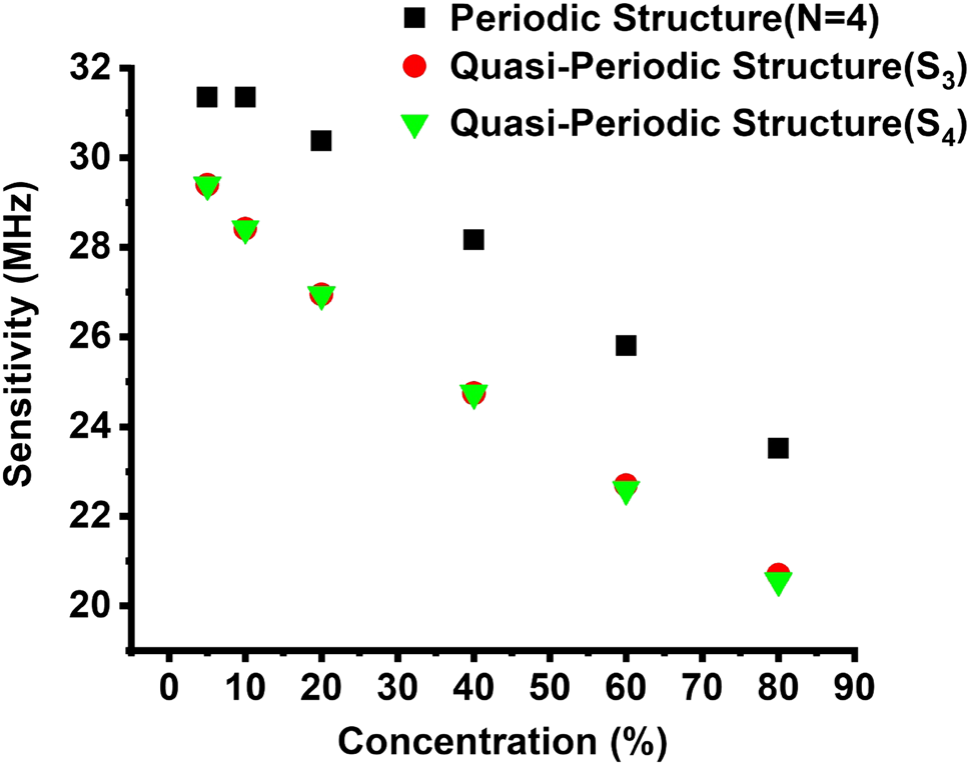
Sensitivity of Periodic PnC structure, S_3_, and S_4_ quasi-periodic PnCs structure to the CO_2_ gas at room temperature as a function of concentration.

As shown in Fig. 5, the periodic/quasi-periodic PnC gas sensor structures’ sensitivity to CO_2_ at room temperature is presented as a concentration function. Equation (20) is used to measure the sensitivity of the proposed PnCs gas sensor structures. The results are displayed in Fig. 5. The highest sensitivity of CO_2_ is provided by the periodic PnC gas sensor structures, as shown in Fig. 5. By comparing these two structures of periodic, as well as the quasi-periodic PnC gas sensor structures, using a concentration value of 10%, the highest sensitivity value of 31.36 MHz was recorded, as shown in Fig. 5 for periodic PnCs gas sensor structures. However, in the same concentration above, a lower sensitivity value of 28.42 MHz was recorded for S_3_ and S_4_ quasi-periodic PnCs gas sensor structures. It is observed that the values of sensitivity are approximately equal in the three PnC gas sensor structures. These values are excellent in the three structures because of the disorder that occurred in these three structures. The acoustic sound waves in the structures underwent substantial attenuation because of the layers number and the arrangement disorder increased. The designed periodic/quasi-periodic PnC gas sensor structures in line with the Fano resonance achieved high sensitivity to the CO_2_ gas. Given the operational frequency of our designed PnC gas sensor in the megahertz range, we require ultrasonic detectors capable of functioning within this frequency range. The impedance analyzer, e.g. two V302-SU (Panametrics, Waltham, MA, USA), Peak frequency 860 kHz, − 6 dB bandwidth 68%, which works well in fluids. Also, Ultrasonic Transducers, such as the Olympus NDT OmniScan MX2, Panasonic UT300C, and Unictron H2KA300KA1CD00 can be used in experimental measurements^92–96^.

### The quality factor, FOM and FWHM of the periodic, S_3,_ and S_4_ quasi-periodic PnC gas sensor structures

**Figure 6:**
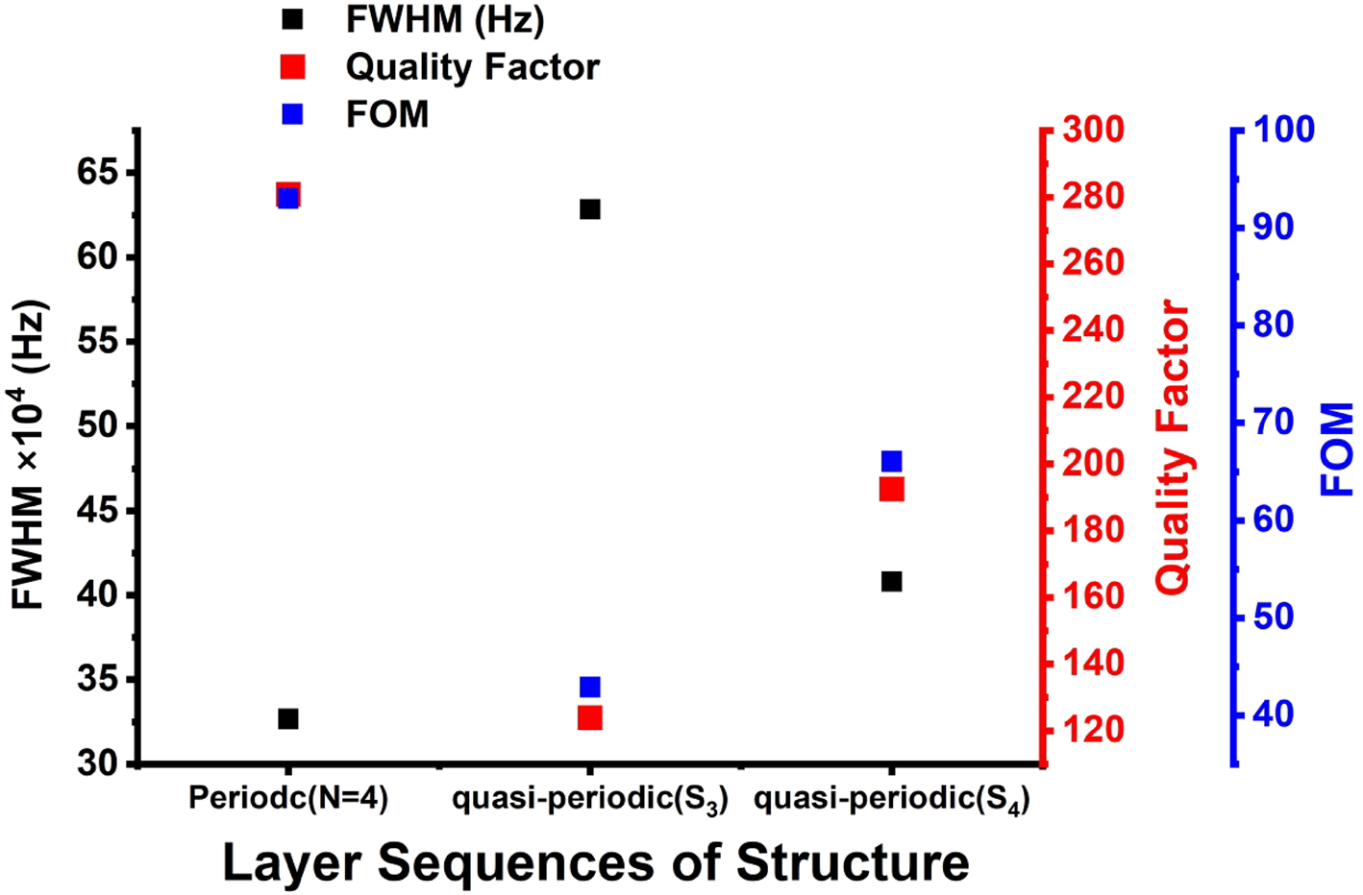
The quality factor, FOM and FWHM of the periodic, S_3,_ and S_4_ quasi-periodic PnC gas sensor structures for CO_2_ sensing in 20% concentration.

The sharp Fano resonance peaks means a resonance peak of high-quality factor and hence a high- performing gas sensor. The values of the quality factor, S, FOM, are obtained using Eqs. (20–22). Thus, the quality factor for periodic, S_3_ and S_4_ quasi-periodic PnCs gas sensor structures is displayed in Fig. 6. The figure shows the highest quality factor is obtained for by periodic, then for S_4_ quasi-periodic PnCs gas sensor structure. Nevertheless, the S_3_ quasi-periodic structure with the layer sequences of [ABADABA] has achieved the lowest quality factor. The periodic design achieved the highest Q value of 280 for at a concentration of 20% followed by S_4_ and S_3_ quasi-periodic designs of about 192, and 123, respectively. The periodic PnCs gas sensor has high Q values because it has a low FWHM in different concentrations, but the S_3_ quasi-periodic PnCs gas sensor has a small Q because its FWHM is greater. Therefore, the FWHM values of the designs are arranged as follows: FWHM_(periodic)_ < FWHM_(S4 quasi-periodic)_ < FWHM_(S3 quasi-periodic)_. The small broadening of the Fano modes is the reason for the high Q values for each CO_2_ peak. The periodic PnC gas sensor has a lower FWHM than the S_4_ quasi-periodic and S_3_ quasi-periodic sensors because the periodic sensor has a more homogeneous structure. The S_4_ and S_3_ quasi-periodic sensors have more disordered structures, which enhances the broadening of the resonant peaks. In addition, it can be attributed to the specific design characteristics and dispersion properties of each structure. In a periodic PnC gas sensor, the periodic arrangement of layers allows for more precise control over the propagation of acoustic waves and the creation of well-defined band gaps. This design optimally interacts with the CO_2_ gas at specific frequencies, leading to a sharper and narrower resonant peak with a smaller FWHM. In contrast, in quasi-periodic PnCs, the arrangement of layers and periodicity affects the propagation of acoustic waves, creating more band gaps and altering the transmission and reflection properties. One difference between S_4_ and S3 quasi-periodic PnC structures is that the large number of layers in S4 results in increasing the acoustic impedance that effect on the displacement of the resonant peak as well. This high impedance and higher number of layers in S4 design over S3 led to small broadening of the Fano modes as well. This configuration optimally interacts with the CO_2_ gas at specific frequencies, leading to higher quality factor (Q) values. The small broadening of the Fano modes in the periodic sensor is the reason for its high Q values. A high Q value means that the sensor is more sensitive to changes in the concentration of CO_2_, which makes it a better gas sensor.

Herein, we examine the impact of the FWHM for Fano resonance peaks of CO_2_ on the Q and FOM of the periodic and S_3_, S_4_ quasi-periodic PnC gas sensor structures. It is generally known that the FWHM of the Fano resonance transmitted peak has an inverse relationship with the PnCs sensor detection accuracy. As can be shown in Fig. 6, FWHM for Fano resonance peak for CO_2_ observed by the periodic structure, the S_3_, and the S_4_ quasi-periodic structures impacted Q and FOM, whereby the maximum Q was obtained for CO_2_ by this periodic structure, and the maximum Fano resonance frequency was obtained for the periodic, as presented in Figs. 3 and 6. However, the lowest Q value was obtained by the S_3_ quasi-periodic structure, as shown in Fig. 6. The FOM of the periodic structure has the highest value of 63 for the periodic followed by the S_4_ quasi-periodic structure, as observed in Fig. 6. The lowest Q and FOM values of 120 and 43 were obtained by the S_3_ quasi-periodic PnCs gas sensor, because of the maximum FWHM value 628.278 kHz, as Q and FOM are inversely connected to FWHM of the Fano resonance transmitted peak, as exemplified in Eqs. (21, 23). Regarding CO_2_, the periodic structure obtained the lowest FWHM value, i.e., 33 kHz (see Fig. 6). The maximum values of Q and FOM were obtained by this periodic PnC structure (i.e., 280 and 93), respectively. According to these results, as shown in Fig. 6, the periodic PnCs structure had the largest FOM when compared to other structures. As a result, the periodic structure had the highest Q and FOM, as shown in Fig. 6. As shown in Fig. 5, the periodic gas sensor obtained the highest sensitivity and resonance frequency to the CO_2_ gas. Based on the obtained results, the periodic structure can introduce an innovative Q and FOM gas sensor to the CO_2_ gas.

### Effect of temperature on the position of Fano mode

**Figure 7:**
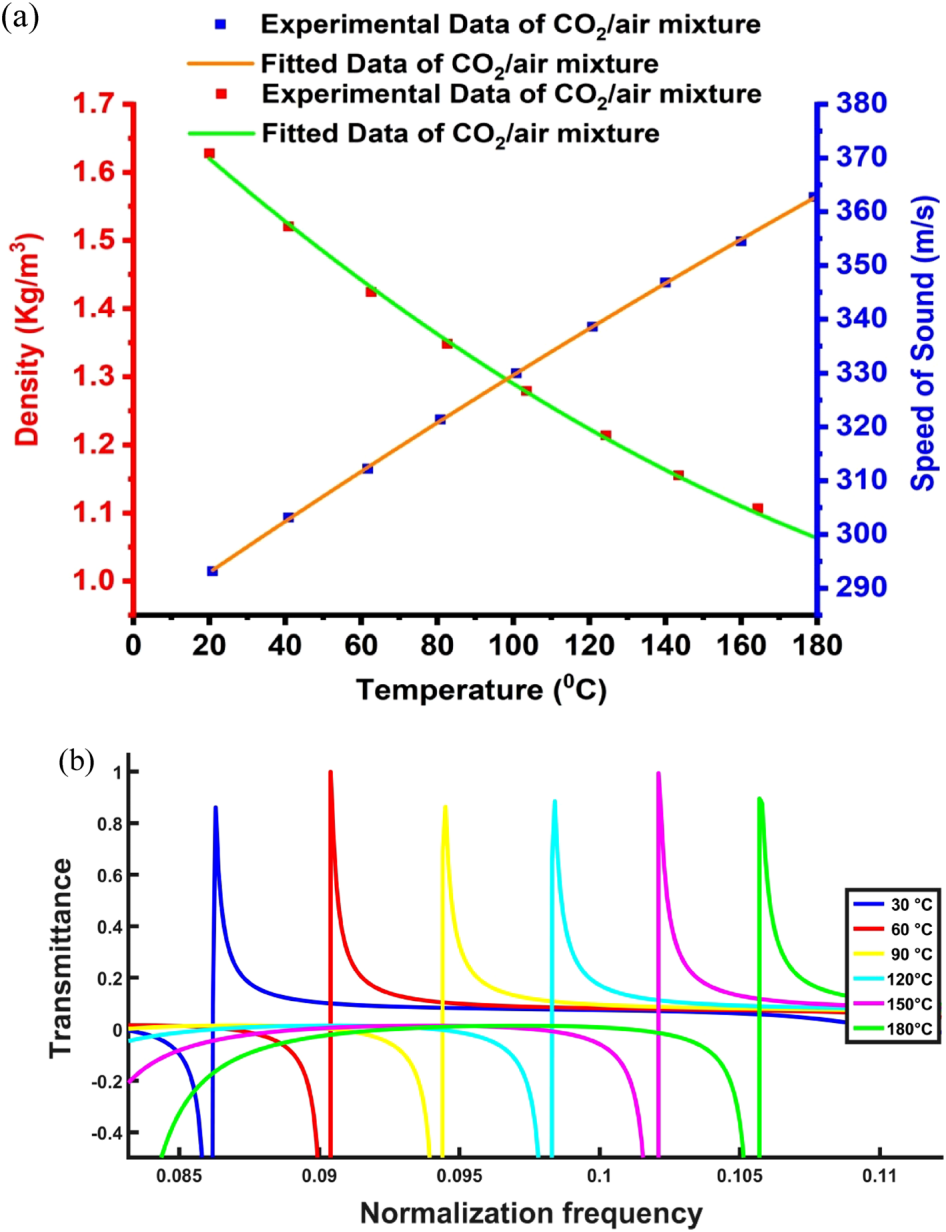
The temperature effect on (**a**) CO_2_ acoustic properties with different temperatures and (**b**) Fano mode position versus temperatures.

According to the results, the used periodic structure represents the most optimum gas sensor structure. It provided higher sensitivity levels and Fano resonance frequency of CO_2_ gas. Additionally, the periodic structure has quality factor and FOM for CO_2_ gas. The sensor may be affected by other environmental factors, for instance, the temperature of the surroundings affects most of sensors. The temperature impact on the mixture’s volume and each gas volume has been calculated depending upon Charles’ Law. Based on this law, the gas temperature at a constant pressure is proportional to the gas volume. This section discusses the effect of different temperatures on the acoustic properties (density and sound velocity) for the periodic gas sensor (see Fig. 7a) based on the experimental data in the provided references. Temperature has a direct impact on the gas density, as well as its acoustic sound speed^97–99^, and with an increased temperature, the gas acoustic sound speed increases, whereas the gas density decreases, as shown in Fig. 7a. As a result, as the temperature increases, the position of CO_2_ Fano resonance peak shifts to a high-frequency band, as illustrated in Fig. 7b. To generalize the correlation between temperatures and acoustic properties, the numerical fitting of the data is studied, and Eq. (24) is used to fit the provided experimental data of the CO_2_ mass density as follows:

24 $$\uprho \left(\frac{\mathrm{kg}}{{\mathrm{m}}^{3}}\right)={\upbeta }_{1}-{\upbeta }_{2}\times \mathrm{T}\left(^\circ \mathrm{C}\right)+{\upbeta }_{3}\times {\left(\mathrm{T}\left(^\circ \mathrm{C}\right)\right)}^{2},$$

where ρ is density, T is temperature, and ${\upbeta }_{1},{\upbeta }_{2} \; {\mathrm{ and}} \; \upbeta _{3}$ represent the fitted relation constants and the values of constants are provided as: ${\upbeta }_{1}=$ 1.71925 $, {\upbeta }_{2}=$ 0.00511 ${\mathrm{and}} \, \upbeta_{3}=$ 8.12149 $\times {10}^{-6}$. As shown in Fig. 7a, there is a decrease in the density of CO_2_ gas with the increase in its temperature; this represents a polynomial linear fitting based on the preceding equation. The gas temperature is connected to the gas density. In other words, the gas density is proportional to the gas temperature. When the CO_2_ temperature increases, the CO_2_ density increases, too. Thus, the experimental data regarding the CO_2_ sound speed are fitted in this equation as follows:

25 $$\mathrm{v}\left(\frac{\mathrm{m}}{\mathrm{s}}\right)={\upbeta }_{1}+{\upbeta }_{2}\times \mathrm{T}\left(^\circ \mathrm{C}\right)-{\upbeta }_{3}\times {\left(\mathrm{T}\left(^\circ \mathrm{C}\right)\right)}^{2},$$

where v refers to the velocity, T is its temperature, and ${\upbeta }_{1},{\upbeta }_{2} \; {\mathrm{ and}} \; \upbeta_{3}$ represent the fitted relation constants. The values of constants are given as:${\upbeta }_{1}=$ 283.27332, ${\upbeta }_{2}=$ 0.49042, ${\mathrm{and}} \; \upbeta_{3}=$ 2.6628 $\times {10}^{-4}$. As shown in Fig. 7a, the sound velocity rises with the rise of CO_2_ temperature, leading to a polynomial linear fit to the preceding equation.

The effect of different temperatures on the position of the Fano resonance peaks of CO_2_ as opposed to the normalized frequency through the periodic gas sensor is presented, as shown in Fig. 7b. In Fig. 7a, different temperatures against the sound speed and density of CO_2_ are debated. Therefore, the effect of these temperatures (30, 60, 90, 120, 150, and 180 °C) on the PnCs structure is considered. Also, it can display the precise quantity of the target CO_2_ gas, as well as the physical characteristics of the gas with remarkable sensitivity, quality, and merit. By considering different temperatures of the CO_2_ gas, the Fano resonance peaks of Fig. 7b will shift to new positions. The frequency and intensity of the Fano resonance peak changes when the CO_2_ temperatures varies, as illustrated in Fig. 7b. The Fano resonance frequency changed from 84 to 88 MHz with changing temperatures within the range between 30 and 60 °C. As observed, the sensitivity recorded 13 MHz when the temperature changes between 30 and 60 °C, respectively. This is because any increase in the CO_2_ temperatures results in an increased CO_2_ density and a decreased sound speed (see Fig. 7a). The Fano resonance peaks’ position changes as well.

### Effect of temperature on the performance of the PnC gas sensor

**Figure 8:**
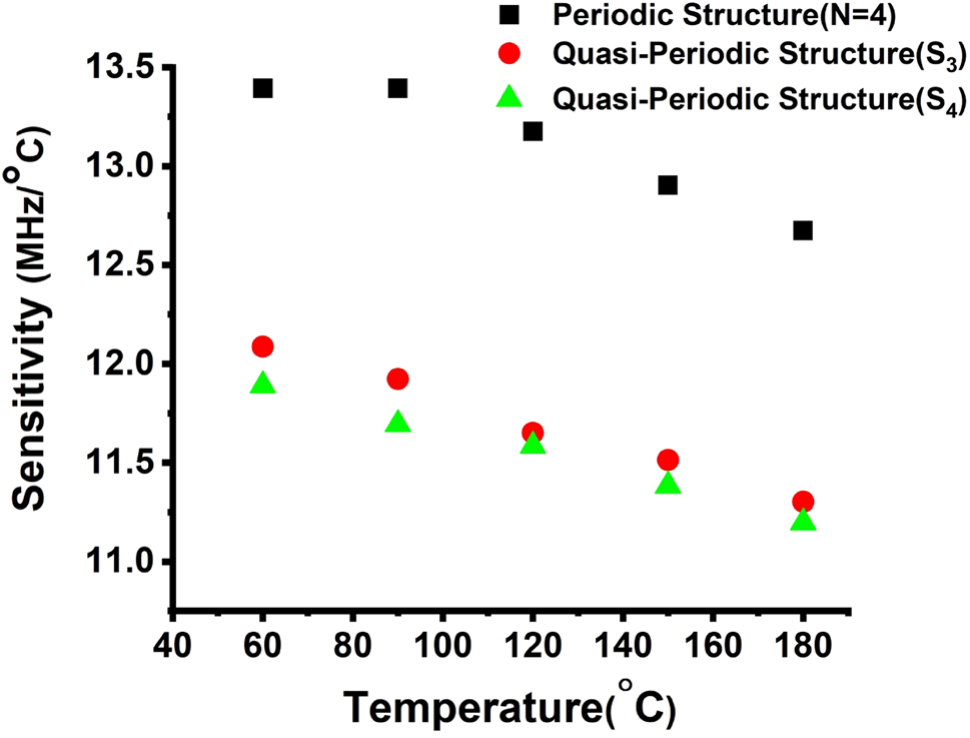
Effect of temperature on the sensitivity of Periodic, S_3_, and S_4_ PnC structures.

This section discusses the periodic and quasi-periodic PnCs gas sensor structures’ sensitivity to CO_2_, introduced as a function of temperature, as presented in Fig. 7b. The periodic gas sensor’s performance is strongly influenced by temperature. It impacts the precision detection. It is the sensor’s capability of detecting the resonance frequency for the sensing medium. When the temperature increases, the sensitivity decreases (see Fig. 8). Based on Eq. (20), the sensitivity of the gas sensor is proportional to Δf_res_, and because of increasing Δf_res,_ the sensitivity increased. Periodic, S_3_, and S_4_ quasi-periodic gas sensors recorded sensitivity for CO_2_ gases with values of 13.4, 12.1, and 11.9 (MHz/°C), respectively at 60 °C, as shown in Fig. 8. Nevertheless, the periodic structure, S_3_ and S_4_ quasi-periodic gas sensors obtained the lowest sensitivity at 180 °C, with the values of 12.5, 11.2, and 11.1 (MHz/°C), respectively. As observed, the three PnCs gas sensor structures obtained distinct sensitivity levels. Because of the disorder that occurred in the three structures, the values of sensitivity are good. These structures experienced significant attenuation of these acoustic sound waves because the number of the layers and the arrangement disorder increased as well. As illustrated in Fig. 8, the designed periodic PnCs gas sensor structure according to the Fano resonance exhibited better sensitivity to the CO_2_ gas.

**Figure 9:**
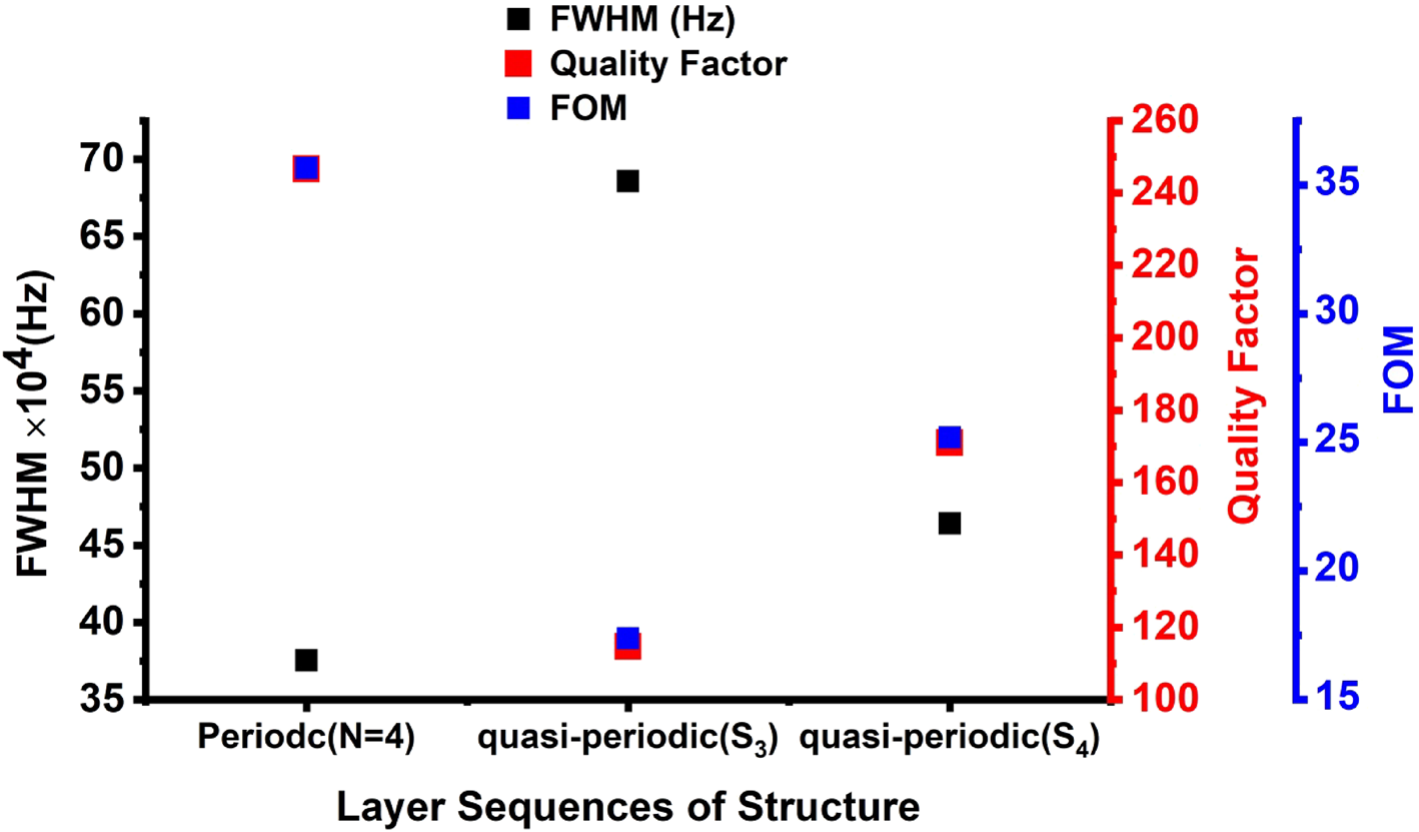
The quality factor, FOM and FWHM of the periodic, S_3_, and S_4_ quasi-periodic PnC gas sensor structures for CO_2_ sensing in 90 °C temperature.

Figure 9 demonstrates the temperature effects on the quality factor and FOM of the periodic, S_3,_ and S_4_ quasi-periodic PnCs gas sensor structures to the CO_2_ gas at different temperatures. The quality factor introduced the sharpness of the Fano resonance peaks, whereby a higher quality factor results in a sharper peak. Figure 9 shows the maximum quality factor and the FOM to the CO_2_ gas values of 246 and 35, respectively in the periodic structure, followed by S_4_ quasi-periodic structure with Q and FOM values of 170 and 25, then the S_4_ quasi-periodic structure with Q and FOM values of 110 and 16, respectively. Thus, the lowest FWHM introduced for the CO_2_ gas at a periodic structure with a value of 37 × 10^4^ Hz followed by S_4_ and S_3_ quasi-periodic structures about 47 × 10^4^ Hz and 67 × 10^4^ Hz, respectively as shown in Fig. 9. Figure 9 shows the highest Q and the FOM at the periodic structure because of the high sensitivity values and the resonance frequency by the periodic gas sensor to the CO_2_ gas (see Fig. 8). Accordingly, the results showed that the periodic structure could introduce an innovative Q and FOM gas sensor to the CO_2_ gas.

### Comparison of the proposed PnC CO_2_ sensor with other sensor designs

This section provides a short comparison between the features of the proposed PnC gas sensor with other PnC gas sensors that may be identical to this design in materials type and dimension. For example, Mehaney theoretically examined the construction of a porous phononic crystal sensor based on a one-dimensional (1D) porous silicon (PSi) phononic crystal (PnCs) sandwiched between two thin rubber layers^59^. Also, Ahmet Cicekh and others presented devices that rely on the evanescent coupling of the surface acoustic waves between two PnCs with trapezoidal grooves on rigid slabs, and they verified them theoretically and experimentally^58^. Moreover, Olgun A. Kaya and other researchers introduced a ring resonator, and they studied the sensor performance experimentally and numerically. The ring resonator uses a one-dimensional PnC on its inner surface^100^. Accordingly, CO_2_ gas development has become increasingly necessary. Therefore, the focus has been placed on this development in the Fano resonance phenomenon because it is a significant phenomenon used for detecting the CO_2_ gas in the surrounding environment.

Additionally, defective PnCs were introduced by Shrouk et al. as a gas sensor that may theoretically detect toxic gases. In another study, Shrouk et al. developed platinum/platinum disulfide (Pt/PtS_2_) composite materials as ultra-sensitive greenhouse gas sensors based on the Fano resonance modes. These materials are based on metal/2D transition metal chalcogenides (TMDs)^72,83^. However, the theoretical gas sensor based on PnC structure used to detect greenhouse gases, including CH_4_, O_2_, CO_2_, and NH_3_, which is introduced by Shrouk et al. needs to be developed to detect the CO_2_-air mixture as the idea of detecting CO_2_ gas in the surrounding atmospheric air is very important, especially for environmental protection.

Moreover, Xiangli et al. studied Fano resonance depending on surface phonon resonance^101,102^. Ting Zhang et al. investigated the occurrence of the Fano resonance mode using a 2D sonic crystal. They measured the Fano resonance peaks created inside the band gap, in addition to the transmission versus frequency^103,104^. Furthermore, Ilyasse et al. investigated the Fano resonance produced by a 1D solid–fluid PnC^101,105^. The previous results encouraged the authors of this paper to demonstrate the designed Fano resonance peaks, which exhibit extremely strong and asymmetric Fano lines, as seen in Figs. 4 and 7b in contrast to previous research.

Accordingly, this study has improved the detection of the CO_2_ gas in the surrounding atmosphere based on the Fano resonance phenomenon-based periodic and quasi-periodic PnCs gas sensor structures, whereby very high resonance transmission modes with an ultra -sensitivity and quality parameters been presented in this research as demonstrated in Table 3.

**Table 3:** A comparison between the sensitivity and quality factor of the design proposed sensor and the results presented in previous literatures.

Sensor design	Sensitivity	Q factor	Reference
Highly efficient gas sensor based on quasi-periodic phononic crystals	0.5988 MHz m^3^ kg^−1^	–	^85^
Temperature influences on the performance of biodiesel phononic crystal sensor	50.37 m^-1^	55.7	^52^
Detection of hazardous greenhouse gases and chemicals with topological edge state using periodically arranged cross-sections	1.58 Hz m^−1^	–	^106^
Modeling of phononic crystal cavity for sensing different biodiesel fuels with high sensitivity	76,589 m^−1^	267	^43^
A promising ultra-sensitive CO_2_ sensor at varying concentrations and temperatures based on Fano resonance phenomenon in different 1D phononic crystal designs	31.5 MHz	280	[The present work]

## Conclusion

This study investigated the performance and efficacy of periodic and Fibonacci quasi-periodic PnC structures for the purpose of CO_2_ sensing applications. This idea of using a PnC design as a gas sensor and especially for the detection of greenhouse gases (e.g. CO_2_) in the air considered as an innovative tool, which focuses on recording the finest displacement of Fano resonance modes through the phononic band gap. By comparing Fibonacci quasi-periodic sequences (S_3_ and S_4_) with the periodic PnC, the periodic structure exhibited the highest performance for CO_2_ detection. The temperature effects were taken into consideration as well. The mathematical formulization of the TMM has been utilized to compute the transmission spectra of PnC designs. The results showed that the highest sensitivity was achieved by the periodic structure with a value of 31.5 MHz. The recorded Q and FOM obtained values of 352 and 110, respectively. The projected sensor in this study has the capability to be reproduced for various gases and liquids. This study provided a simple sensor for determining the levels of CO_2_ in the air with very high accuracy. Where, the concentration sensitivity reached the value of 31.5 MHz. The proposed sensor has other advantages. These include the ease of construction, cost-effectiveness, and readily available materials without the use of electronic components (Supplementary Table S1).

### Supplementary Information

Supplementary Table S1.

## Data Availability

Requests should be addressed to corresponding author.
