# Supplementary material for: A promising ultra-sensitive CO2 sensor at varying concentrations and temperatures based on Fano resonance phenomenon in different 1D phononic crystal designs
Source: Sci Rep. 2023 Sep 12;13:15028. doi: 10.1038/s41598-023-41999-1 (PMC10497549; doi:10.1038/s41598-023-41999-1)
Supplement: Supplementary file 1 — Supplementary Table S1. [file 41598_2023_41999_MOESM1_ESM.docx]

Table S1: Sensitivity comparison of Different PnC Structures with Varying Numbers of Layers in case 80% concentration.

| Type of Structure (S_n_) | Number of layers | Sensitivity |
| --- | --- | --- |
| Periodic PnC Structure | 11 | 23520000 Hz |
| S_3_ PnC Structure | 7 | 20702500 Hz |
| S_4_ PnC Structure | 11 | 20567750 Hz |
| S_5_ PnC Structure | 17 | 980000 Hz |
| S_6_ PnC Structure | 27 | 490000 Hz |
